# Renal hemosiderosis presenting with acute kidney Injury and macroscopic hematuria in Immunoglobulin A nephropathy: a case report

**DOI:** 10.1186/s12882-021-02334-w

**Published:** 2021-04-15

**Authors:** Shinya Taguchi, Sumi Hidaka, Mitsuru Yanai, Kunihiro Ishioka, Kenji Matsui, Yasuhiro Mochida, Hidekazu Moriya, Takayasu Ohtake, Shuzo Kobayashi

**Affiliations:** 1 Kidney Disease and Transplant Center, Shonan Kamakura General Hospital, 1370-1 Okamoto, 247-8553 Kamakura, Kanagawa Japan; 2 Department of Medical Science and Cardiorenal Medicine, Yokohama City University Graduate School of Medicine, 3-9 Fukuura, Kanazawa-ku, 236-0004 Yokohama, Japan; 3 Department of Pathology, Sapporo Tokushukai Hospital, 1-1-1 Oyachi-higashi, Atsubetsu-ku, 004-0041 Sapporo, Hokkaido Japan; 4 Hokkaido Renal Pathology Center, IT-FRONTBuilding, 28-196, N9W15, Chuo-ku, 060-0009 Sapporo, Hokkaido Japan

**Keywords:** IgA nephropathy, Macroscopic hematuria, Macrohematuria, Immunoglobulin A, Hemosiderin, Hemosiderosis, Acute kidney injury, Acute tubular necrosis

## Abstract

**Background:**

Macroscopic hematuria-associated acute kidney injury (AKI) is a well-known complication of immunoglobulin A (IgA) nephropathy. In such cases, intratubular obstruction by red blood cell (RBC) casts and acute tubular necrosis are mainly observed pathologically. Herein, we report the case of a patient with IgA nephropathy presenting with AKI following an episode of macrohematuria. The patient presented with severe renal tubular hemosiderosis and acute tubular necrosis and without any obvious obstructive RBC casts.

**Case presentation:**

A 68-year-old woman, who was diagnosed with IgA nephropathy on renal biopsy 6 years ago, was admitted to our hospital after an episode of macroscopic glomerular hematuria and AKI following upper respiratory tract infection. Renal biopsy showed mesangial proliferation of the glomeruli, including crescent formation in 17 % of the glomeruli, and acute tubular necrosis without obvious hemorrhage or obstructive RBC casts. The application of Perls’ Prussian blue stain showed hemosiderin deposition in the renal proximal tubular cells. Immunofluorescence showed granular mesangial deposits of IgA and C3. Based on these findings, she was diagnosed with acute tubular necrosis with a concurrent IgA nephropathy flare-up. Moreover, direct tubular injury by heme and iron was considered to be the cause of AKI. She was treated with intravenous pulse methylprednisolone followed by oral prednisolone. Thereafter, the gross hematuria gradually faded, and her serum creatinine levels decreased.

**Conclusions:**

IgA nephropathy presenting with acute kidney injury accompanied by macrohematuria may cause renal hemosiderosis and acute tubular necrosis without obstructive RBC casts. Hemosiderosis may be a useful indicator for determining the pathophysiology of macroscopic hematuria-associated AKI. However, renal hemosiderosis may remain undiagnosed. Thus, Perls’ Prussian blue iron staining should be more widely used in patients presenting with hematuria.

## Background

Macroscopic hematuria-associated acute kidney injury (AKI) is a well-known complication of immunoglobulin A (IgA) nephropathy (IgAN) [1–3]. In such cases, intratubular red blood cell (RBC) casts and acute tubular injury are mainly observed pathologically [1–3].Additionally, deposition of hemosiderin in renal tubules has been reported in cases of paroxysmal nocturnal hemoglobinuria (PNH) [4] and anticoagulant-related nephropathy (ARN) [5] presenting with AKI accompanied by macrohematuria. Herein, we report the case of a patient with IgAN presenting with AKI accompanied by macrohematuria. The patient was presented with severe renal tubular hemosiderosis and acute tubular necrosis (ATN) without obvious obstructive RBC casts. In this case, renal hemosiderosis was expected to be the cause of the AKI.

## Case presentation

A 69-year-old woman was admitted to our hospital with a 5-day history of macroscopic hematuria and AKI. She was diagnosed with IgAN on renal biopsy 6 years prior. Following this diagnosis, she was administered prednisolone because she had persistent microscopic hematuria and proteinuria of approximately 1.0 g/day. Proteinuria remitted after treatment; however, microscopic hematuria persisted (over 100 RBCs per high power field). Prednisolone was discontinued 1 year ago. Five days prior to admission, she developed an upper respiratory tract infection, accompanied by gross hematuria. The gross hematuria persisted; however, the color became lighter after admission. Her past medical history included hypertension, diabetes, and dyslipidemia. She was not receiving any antithrombotic medication. On admission, her body temperature, blood pressure, and heart rate were 36.6℃, 144/64 mmHg, and 65 beats/min, respectively. She had no abnormal findings on examination of her tonsils, pharynx, extremities, and skin. Laboratory data are presented in Table 1. Her serum creatinine level increased to 4.9 mg/dl from 1.1 mg/dl at the last visit. Urinalysis revealed that the urinary protein excretion level was 1.25 g/day; the sediments contained 30–49 RBCs and ≥ 100 white blood cells per high-power field. There were no signs of dehydration. Abdominal ultrasound showed that both kidneys were of normal size, excluding hydronephrosis.

**Table 1:** Laboratory data on admission

Parameter	Value (reference range)
Hematology	
White blood cell count, /µL	7400 (3000–9700)
Hemoglobin, mg/dL	12.6 (11.0–15.6)
Platelet count, 10^4^/µL	29.7 (12.4–30.5)
Blood chemistry	
Total protein, g/dL	7.4 (6.4–8.3)
Albumin, g/dL	3.4 (3.8–5.2)
Urea nitrogen, mg/dL	62.1 (7.4–19.5)
Creatinine, mg/dL	4.92 (0.4–1.0)
eGFR, ml/min/1.73m^2^	7.5 (> 60)
AST, U/L	23 (12–35)
ALT, U/L	24 (6–40)
LDH, U/L	229 (119–229)
FBS, mg/dL	143 (70–110)
HbA1c, %	6.8 (4.6–6.2)
Triglyceride, mg/dL	169 (50–149)
LDL cholesterol, mg/dL	146 (70–139)
Immunology	
IgG, mg/dL	1389 (820–1740)
IgA, mg/dL	736 (90–400)
IgM, mg/dL	67 (52–270)
C3, mg/dL	157 (80–140)
C4, mg/dL	42 (11–34)
CH50, U/mL	52 (30–45)
C-reaction protein, mg/dL	6.31 (< 0.5)
Antinuclear antibody, IU/mL	×40 (< 40)
Anti-DNA antibody, IU/mL	< 2.0 (0–6.0)
MPO-ANCA, U/mL	< 1.0 (0–3.5)
PR3-ANCA, U/mL	< 1.0 (0–3.5)
ASO, IU/mL	50 (0–239)
Urinalysis	
Occult blood	3+
Dipstick protein	2+
Red blood cell, /HPF	30–49 (< 5)
White blood cell, /HPF	> 100 (< 5)
Protein urea, g/g creatinine	1.25 (< 0.15)
Red blood cell cast	(-)

*eGFR* estimated glomerular filtration rate; *AST* aspartate aminotransferase; *ALT* alanine aminotransferase; *LDH* lactate dehydrogenase; *FBS* fasting blood sugar; *HbA1c* hemoglobin A1c; *LDL* low density lipoprotein; *Ig* immunoglobulin; *MPO* myeloperoxidase; *PR3* proteinase 3; *ANCA* anti-neutrophil cytoplasmic antibody; *ASO* anti-streptolysin

**Fig. 1:**
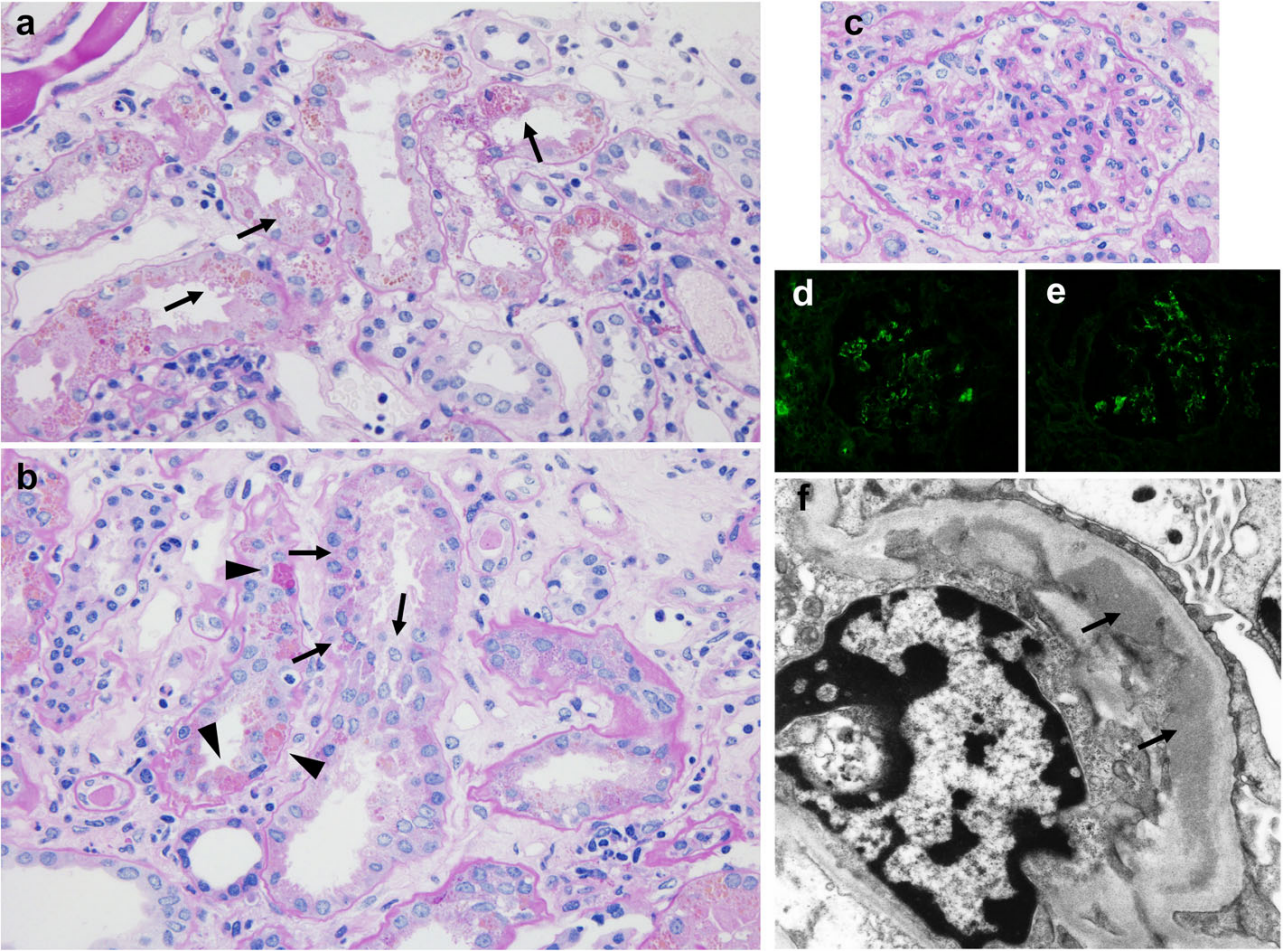
Microscopic findings of the renal biopsy. Periodic acid-Schiff staining shows tubular necrosis and pigments of various colors ranging from yellow to brown in the proximal tubular epithelial cells (**a**: ×400, **b**: ×400). **a** Black arrow shows loss of the brush border and swelling of the tubular epithelium. **b** Black arrow shows detachment of tubular epithelium from the basement membrane. Black arrowhead shows coagulative necrosis of the tubular epithelium. **c** Mesangial proliferation in the glomeruli (**c**: ×400). Immunofluorescence shows granular mesangial staining for immunoglobulin A (IgA) (**d**) and C3 (**e**), without staining for IgG, IgM, and C1q. Electron microscopy shows para-mesangial electron-dense deposits (**f**: ×8000, black arrow)

**Fig. 2:**
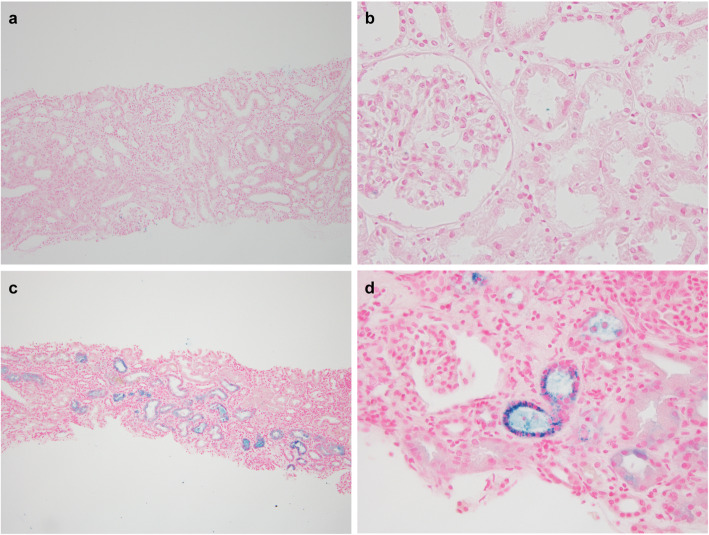
Perls’ Prussian blue staining. The first kidney biopsy performed 6 years ago was negative for Perls’ Prussian blue staining (**a**: ×100, **b**: ×1000). However, the second kidney biopsy, which was performed for the purposes of this work, showed diffuse hemosiderin deposits in the proximal tubular epithelial cells (**c**: ×100, **d**: ×1000)

**Fig. 3:**
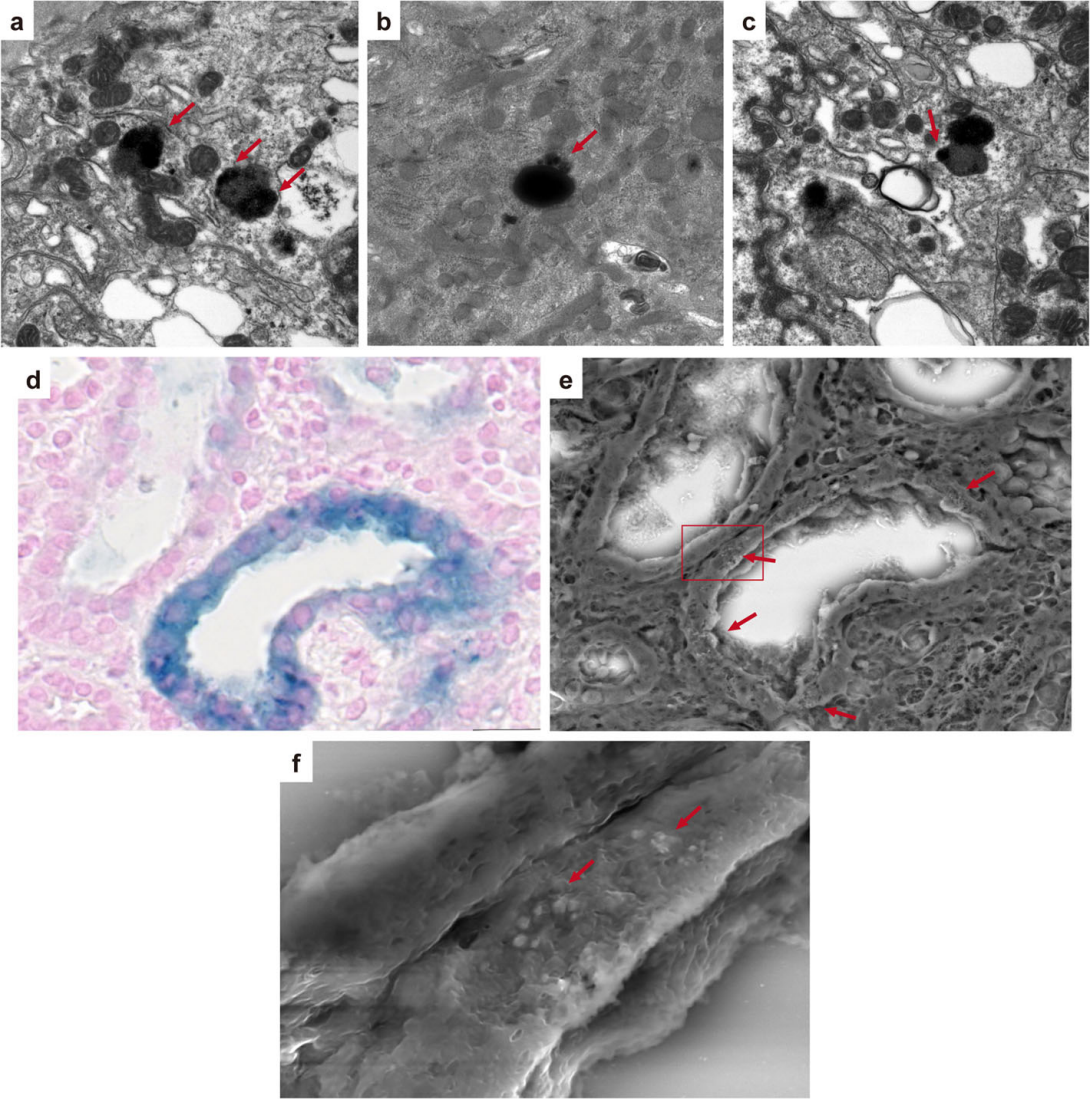
Electron microscopy of renal biopsied specimen. Transmission electron microscopy shows electron-dense particles in lysosomes of tubular epithelial cells (red arrow, **a**: ×8000, **b**: ×8000, **c**: ×8000). **d**, **e** Light microscopy of Perls’ Prussian blue staining and low vacuum scanning electron microscopy images of the same area, respectively (**d**: ×1000, **e**: ×1000). **e** Red arrows show white electron-dense particles, corresponding to the blue granules in **d**. Regarding the tubular cells that are not stained blue in **d**, no electron-dense particles are observed in **e**. **f** shows higher magnification image of the red square area in **e** (**f**: ×6000). The red arrows indicate electron-dense particles

A renal biopsy was performed 7 days after admission. Light microscopy showed that nine out of 29 (31 %) glomeruli were globally sclerotic, and five out of 29 (17 %) had cellular crescents. Most glomeruli showed moderate mesangial proliferation. The proximal tubule epithelial cells were edematous, showed detachment from the tubular basement membrane, and contained granules of various colors (ranging from yellow to brown). Some tubules showed dilation and loss of the brush border. There was no obvious hemorrhage or obstructive RBC casts in the tubules. There was interstitial fibrosis and inflammatory cells were present around the injured tubules (Fig. 1a–c). Immunofluorescence showed granular mesangial staining for IgA and C3 (Fig. 1d, e). Electron microscopy showed mesangial electron-dense deposits (Fig. 1 g). Perls’ Prussian blue staining showed hemosiderin deposition in the renal proximal tubular cells, which was not observed 6 years ago when first renal biopsy was performed (Fig. 2). Low vacuum scanning electron microscopy, a novel method for rapid three-dimensional pathological analysis [6, 7], showed the electron-dense particles in tubular epithelial cells. Furthermore, transmission electron microscopy revealed that these particles were located in lysosomes (Fig. 3). Based on these findings, she was diagnosed with ATN with a concurrent IgAN flare-up. After confirming the results of the renal biopsy, she was treated with intravenous pulse methylprednisolone, followed by oral prednisolone. The gross hematuria gradually faded, and the serum creatinine levels decreased. After 3 months, the serum creatinine and urinary protein excretion levels were 1.8 mg/dl and 0.24 g/day, respectively.

## Discussion and conclusions

The patient in the present case was diagnosed with IgAN 6 years ago. She presented with AKI and macroscopic hematuria after upper respiratory tract infection. Renal biopsy specimens showed severe renal tubular hemosiderosis and ATN without obvious obstructive RBC casts, which had never been reported previously. This case suggests that hemosiderin-induced tubulotoxicity can be a major cause of AKI.

In this case, there were no obvious obstructive RBC casts in the tubules, which is commonly observed in patients with macroscopic hematuria-associated AKI [8]; however, significant hemosiderosis in the tubules was observed. Initially, it was suggested that intratubular obstruction by RBCs or hemoglobin casts induced the AKI and macrohematuria [1]; however, diverse mechanisms of tubular injury have been proposed recently. Hemoglobin, an intratubular degradation product of erythrocytes, is often taken up by proximal tubular epithelial cells. Hemoglobin, heme, and iron are degraded and accumulate in the tubular cells, causing tubular injury through oxidative stress, inflammation, mitochondrial damage, and fibrosis [8, 9]. It has also been suggested that the heme group of the hemoglobin molecule promotes intrarenal vasoconstriction and podocyte injury [8, 10]. Moreover, the iron that cannot be processed as ferritin in the tubular cells, due to excessive hemoglobin exposure, accumulates as hemosiderin [11]. Tubular hemosiderosis observed in our case indicated that there was excess iron in the renal tubules, suggesting that the acute tubular injury was caused by iron and other heme-containing molecules. It was reported that < 25 % of glomeruli were affected by crescents, which were characteristically small, cellular, and segmental in all cases of IgAN that developed into AKI following macrohematuria [3]. In our case, only 17 % of the glomeruli showed crescents, in contrast to the diffuse severe ATN. Taking these pathological findings into account, we could not explain the AKI cause by these glomerular changes. Thus, in this case, the AKI cause was considered to be ATN induced by tubulotoxicity, which was caused by iron and heme-containing molecules.

Our patient was treated with steroid therapy because massive microhematuria, which represents glomerular inflammation and could result in tubular injury, was persisting. Previous studies have demonstrated that the duration of gross hematuria correlates with serum creatinine at the last observation in macroscopic hematuria-associated AKI [3]. In addition, 38.9 % of hematuria-associated AKI episodes were treated with steroids, and the duration of hematuria was shortened [3]. However, there have been no prospective studies examining these results, and the effect of steroids on macroscopic hematuria-associated AKI, including ATN due to the hemosiderin deposition, is inconclusive. Therefore, the validity of steroid treatment for this case needs to be determined by accumulating evidence in the future.

Hemosiderin deposition in tubules has been reported in various diseases with intravascular hemolysis, including PNH [4], sickle cell anemia [12], a prosthetic heart valve replacement complication [13], and in a few cases of diseases with glomerular hemorrhage including ARN [5] and IgAN [8]. A previous work reported that approximately 25 % of patients with PNH presented with macroscopic hemoglobinuria [14]; however, Clark et al., in their autopsy study including seven cadavers with PNH, showed that all cases had moderate to severe hemosiderin deposition in the proximal tubules [15]. In the present case, the microscopic hematuria persisted after the diagnosis of IgAN 6 years ago, and the time phase of hemosiderin deposition was varied, suggesting that hemosiderin had been formed prior to her current presentation. Thus, renal hemosiderosis may have been caused by macroscopic hematuria and by the persistent microscopic hematuria. It was also suggested that renal hemosiderosis might be a tubular injury marker caused by hematuria, whether macroscopic or microscopic. However, renal hemosiderosis may remain unrecognized, as hemosiderin is difficult to be identified without Perls’ Prussian Blue iron staining; thus, this should be more widely used in cases presenting with hematuria.

Initially, hematuria was considered to be a benign manifestation of renal disease [16], and the association between hematuria and renal outcomes has not yet been fully evaluated. However, poor renal outcomes associated with hematuria have recently been reported [3, 17]. A study evaluating the outcomes of patients who had IgAN presenting AKI and macrohematuria has shown that up to 25 % of patients do not return to their baseline renal function [3]. In an epidemiological study, persistent asymptomatic isolated microhematuria was significantly associated with an increased risk of end-stage renal disease after 22 years of follow-up examination in 1 million young Israeli participants [17]. Taking iron and heme toxicities into account, the association between hematuria and worsening renal outcomes might be mediated by tubular injury after oxidative stress and inflammation due to iron and heme. The pathological predictors of renal outcomes, currently reported in cases of IgAN, were segmental glomerulosclerosis, tubular atrophy, and interstitial fibrosis [18]. Future studies are required to evaluate the prevalence of renal hemosiderosis in diseases that present with hematuria, including IgAN, and to examine the correlation between hemosiderosis and renal outcomes.

In conclusion, IgAN presenting with AKI accompanied by macrohematuria, can present with renal hemosiderosis and ATN without obstructive RBC casts. Moreover, hemosiderosis may be a useful indicator for determining the pathophysiology of macroscopic hematuria-associated AKI. However, renal hemosiderosis may remain undiagnosed, since hemosiderin is difficult to identify without Perls’ Prussian Blue iron staining. Additionally, the clinical sequelae of renal hemosiderosis and its effects on renal prognosis remain unclear. Perls’ Prussian Blue iron staining should be more widely used in cases presenting with hematuria. Future studies should evaluate the role of renal hemosiderosis on prognosis and determine whether it should be used as a prognostic factor of renal outcomes.

## Data Availability

All data generated or analyzed during this study are included in this published article.

## Abbreviations

AKI: Acute kidney injury
PNH: Paroxysmal nocturnal hemoglobinuria
ARN: Anticoagulant-related nephropathy
IgAN: Immunoglobulin A nephropathy
RBC: Red blood cell
ATN: Acute tubular necrosis
